# Efficient Delivery of SARS-CoV-2 Plasmid DNA in HEK-293T Cells Using Chitosan Nanoparticles

**DOI:** 10.3390/ph18050683

**Published:** 2025-05-05

**Authors:** Citlali Cecilia Mendoza-Guevara, Alejandro Martinez-Escobar, María del Pilar Ramos-Godínez, José Esteban Muñoz-Medina, Eva Ramon-Gallegos

**Affiliations:** 1 Laboratorio de Citopatología Ambiental, Departamento de Morfología, Escuela Nacional de Ciencias Biológicas, Instituto Politécnico Nacional (IPN), Ciudad de México 07738, Mexico; ccmg.vet@gmail.com (C.C.M.-G.); ledcano_89@hotmail.com (A.M.-E.); 2 Laboratorio de Microscopía Electrónica, Instituto Nacional de Cancerología (INCAN), Ciudad de México 14080, Mexico; pilyrg@gmail.com; 3 Banco de Muestras de la CCILE, Instituto Mexicano del Seguro Social (IMSS), Ciudad de México 07760, Mexico; jose.munozm@imss.gob.mx

**Keywords:** gene therapy, nanovehicle, nanotechnology, polyplexes, transfection

## Abstract

**Background/Objectives**: Gene therapy has emerged as a promising strategy for treating a wide range of diseases. However, a major challenge remains in developing efficient and safe delivery systems for genetic material. Nanoparticles, particularly chitosan nanoparticles (CNPs), have gained significant attention as a potential solution. This study focuses on designing a SARS-CoV-2 plasmid DNA (pDNA) conjugated with CNPs and evaluating its in vitro delivery efficiency. **Methods**: The Omicron Spike DNA sequence was inserted into the pIRES2-eGFP expression vector, and CNPs were synthesized with optimized physicochemical properties to enhance stability, cellular uptake, and transfection efficiency. The conjugate was characterized using UV-Vis, FT-IR, DLS, and TEM techniques. Transfection efficiency was assessed and compared to the commercially available TurboFect reagent as a control. **Results**: CNPs-pDNA polyplexes with an average size of 159.0 ± 33.1 nm (TEM), a zeta potential of +19.7 ± 0.3 mV, and 100% ± 0.0 encapsulation efficiency were developed as a non-viral delivery system. CNPs efficiently serve as a delivery vehicle for the constructed pDNA without altering cell morphology, achieving transfection efficiencies of 62–74%, compared to 55–70% for TurboFect. Furthermore, RT-qPCR confirmed the expression of Spike mRNA, and Western blot assays validated the expression of Spike protein. Notably, Spike protein expression from CNPs was found to be two-fold higher than the control at 96 h post-transfection. **Conclusions**: These findings suggest that CNPs are a promising and versatile platform for delivering genetic material. Importantly, this study highlights the intrinsic properties of chitosan, without the use of additional ligands, as a key factor in achieving efficient gene delivery.

## 1. Introduction

Gene therapy and vaccine development have emerged as critical areas of intense research in addressing genetic disorders, infectious diseases, and other medical conditions [1,2]. A significant challenge in these fields lies in the development of efficient, safe, and targeted delivery systems for genetic material. Conventional methods, such as viral vectors, have shown high transfection efficiency but are often limited by safety concerns, including immunogenicity and insertional mutagenesis [3,4]. In this context, non-viral delivery systems, particularly nanoparticles, have emerged as promising tools for gene delivery and vaccine development, offering numerous advantages over conventional approaches [5,6].

Nanoparticles, owing to their subcellular size, can efficiently navigate through biological tissues and penetrate barriers such as cell membranes and the blood–brain barrier [7,8]. They also offer protection against nucleases, enhance cellular uptake, and minimize off-target effects, making them promising candidates for gene delivery and vaccine development [4,8,9]. Among the various nanomaterials explored, chitosan nanoparticles (CNPs) have garnered significant interest due to their unique properties and potential applications in biomedicine [10,11,12].

Chitosan (Chi) is a natural, cationic polysaccharide derived from the shells of crustaceans (e.g., shrimps and crabs) and the cell walls of fungi. It is biocompatible, biodegradable, mucoadhesive, and has been approved by the FDA for use in tissue engineering and drug delivery [13,14]. CNPs can form polyplexes with DNA through coacervation, driven by ionic interactions between the positively charged chitosan and the negatively charged nucleic acids [15,16]. These polyplexes can be internalized by cells via endocytosis, facilitated by electrostatic interactions with the cell membrane, and can escape endosomal degradation, enabling efficient gene delivery [16,17]. Additionally, CNPs can be phagocytosed by macrophages, which may influence their distribution and delivery to specific tissues [18].

Compared to other cationic polymers commonly used in gene delivery, such as polyethyleneimine (PEI) and liposomes, chitosan offers a favorable safety profile, practical formulation advantages, and intrinsic immunostimulatory effects. While PEI is valued for its high transfection efficiency, its cytotoxicity and poor biodegradability limit its clinical applicability [19]. Liposomes, though versatile, require complex formulation and offer lower mucoadhesive capacity [20]. In contrast, chitosan’s natural mucoadhesion promotes prolonged residence time at mucosal sites, and its ability to stimulate immune responses makes it particularly attractive for vaccine delivery [21].

Chitosan also promotes endosomal escape via the “proton sponge” effect, similar to PEI, enhancing the intracellular release of genetic material [22]. Its ability to interact with toll-like receptors and stimulate macrophage and dendritic cell activation has further positioned it as a potential vaccine adjuvant [23].

Despite their advantages, CNPs face several limitations that hinder their widespread application. These limitations include poor solubility at physiological pH, batch-to-batch variability in molecular weight and degree of deacetylation, and relatively lower transfection efficiency compared to viral vectors due to limited endosomal escape [4,24]. To overcome these issues, evidence suggests that improving nanoparticle stability, solubility, and endosomal escape is critical [25].

Furthermore, since the size and surface charge of CNPs can significantly impact their cellular uptake and biodistribution, with larger particles or excessive positive charge leading to aggregation or cytotoxicity [26,27], strategies such as surface modification with targeting ligands, incorporation of endosomolytic agents, and optimization of nanoparticle synthesis parameters have emerged [28,29]. However, these modifications or hybrid systems often complicate synthesis, increase production costs, and introduce regulatory hurdles.

In this study, we address these challenges by designing and synthesizing chitosan nanoparticles with optimized physicochemical properties for efficient gene delivery. We developed a recombinant plasmid encoding the SARS-CoV-2 Spike protein and formed CNPs-pDNA polyplexes to evaluate their in vitro delivery effectiveness. Our approach incorporates recent advancements in nanoparticle engineering to enhance stability, cellular uptake, and transfection efficiency. While many studies incorporate ligands to enhance delivery, our study focuses solely on the intrinsic properties of chitosan, without the use of additional ligands, offering a simpler and more cost-effective alternative while achieving adequate transfection efficiencies.

## 2. Results

### 2.1. Construction of DNA Plasmid pIRES2-eGFP-Spike

**Figure 1:**
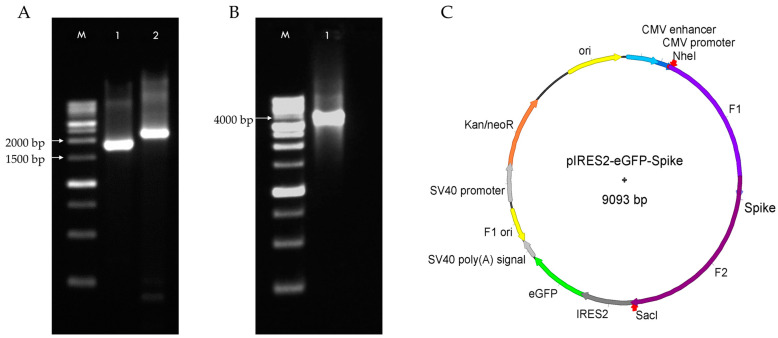
Obtention of the SARS-CoV-2 Spike sequence and construction of the pIRES2-eGFP-Spike plasmid. (**A**) Gel electrophoresis of Spike gene fragments with recognition sites for NheI and SacI, obtained by RT-PCR. Lane M: 1 kb DNA ladder (GOLDBIO, D010-500); Lane 1: Spike fragment F1 (1986 bp); and Lane 2: Spike fragment F2 (2208 bp). (**B**) Gel electrophoresis of the full-length Spike sequence (3843 bp) assembled by POE-T. Lane M: 1 kb ladder (GOLDBIO, D010-500); Lane 1: assembled product (F1 + F2). (**C**) Schematic representation of the pIRES2-eGFP-Spike plasmid (9093 bp), showing the insertion of the full-length SARS-CoV-2 Spike sequence (3789 bp) between the NheI and SacI restriction sites of the pIRES2-eGFP expression vector.

The recombinant plasmid DNA containing the Spike sequence was constructed in two phases. First, the Spike gene was amplified in two overlapping fragments (F1 and F2) by RT-PCR from RNA samples positive for SARS-CoV-2. The expected sizes of the PCR products (F1: 1986 bp and F2: 2208 bp) were confirmed by agarose gel electrophoresis (Figure 1A). Next, the full-length Spike sequence (3843 bp) was assembled using the touchdown Polymerase Overlap Extension (POE-T) and verified by electrophoresis (Figure 1B). The purified full-length Spike amplicon was then cloned into the pIRES2-eGFP expression vector used as the backbone, resulting in the recombinant plasmid pIRES2-eGFP-Spike. The correct insertion was validated by restriction analysis and sequencing of the CMV–Spike region. A schematic plasmid map based on the sequencing results is shown in Figure 1C.

**Figure 2:**
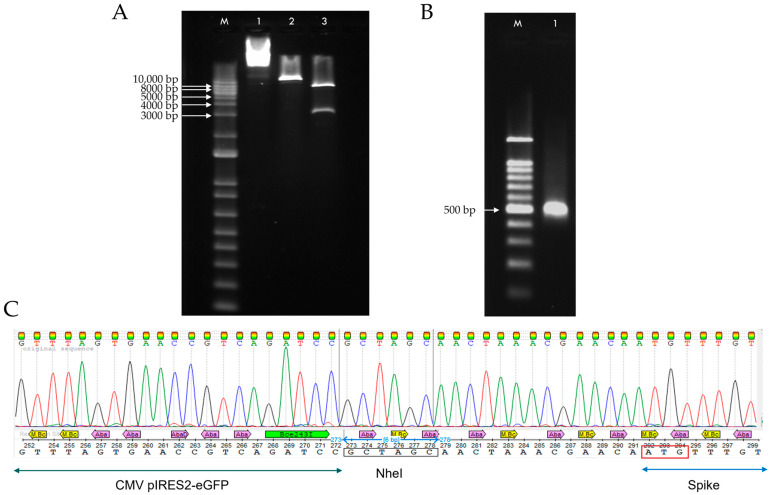
Validation of recombinant plasmid pIRES2-eGFP-Spike. (**A**) Restriction digestion analysis of pIRES2-eGFP-Spike plasmid with NheI and SacI. M: 1 kb ladder (Invitrogen, 10787018); Lane 1: non-digested plasmid; Lane 2: plasmid digested with SacI (linearized, 9093 bp); Lane 3: double digestion with NheI and SacI releasing two bands corresponding to the pIRES2-eGFP backbone (5304 bp) and the Spike insert (3789 bp). (**B**) PCR amplification of the CMV–Spike region of pIRES2-eGFP-Spike. M: 100 bp ladder (GOLDBIO, D001-500); Lane 1: CMV–Spike PCR product (397 bp). (**C**) Sanger sequencing chromatogram of the CMV–Spike PCR product showing the NheI recognition site (black box) and the Spike start codon (red box).

Validation of the recombinant plasmid pIRES2-eGFP-Spike was performed by restriction digestion using NheI and SacI, which released two fragments of the expected sizes, 5279 bp and 3826 bp, corresponding to the pIRES2-eGFP vector and the Spike insert, respectively (Figure 2A). Further confirmation was obtained by PCR using primers flanking the CMV promoter and the 5′ region of the Spike gene, which amplified a 397 bp fragment (Figure 2B). The identity of the amplified region was confirmed by Sanger sequencing. The chromatogram confirmed the expected sequence corresponding to the CMV–Spike junction, including the NheI restriction site and the Spike start codon (Figure 2C).

### 2.2. Retardation and Release Assay of CNPs-pDNA Polyplexes

**Figure 3:**
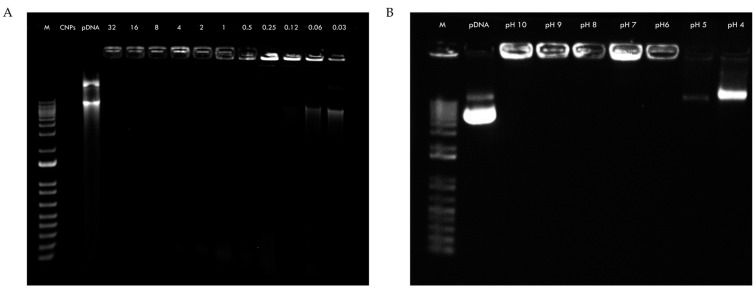
Retention and release agarose gel electrophoresis of CNPs-pDNA complexes. (**A**) Gel electrophoresis showing the retention of pDNA at different C/P weight ratios (32:1–0.03:1). (**B**) Release assay of polyplexes incubated in DMEM at different pH values (pH 10–4), with pDNA release observed at lower pH values. M: 1 kb ladder (Invitrogen, 10787018); CNPs: chitosan nanoparticles; pDNA: plasmid DNA.

The binding efficiency of pDNA to CNPs was assessed by a gel retardation assay. Naked pDNA and CNPs were used as controls to observe their normal migration. Naked CNPs did not interact with SYBR^TM^ Gold; thus, no signal was detected. In contrast, most polyplexes at various C/P ratios showed a complete retardation effect, indicating successful binding between pDNA and CNPs (except at 0.12:1, 0.06:1, and 0.03:1 ratios) (Figure 3A). To quantify the binding efficiency, the polyplexes were centrifuged and the amount of unbound pDNA in the supernatant was measured. The results showed a binding efficiency of 100% ± 0.0 for most of the C/P ratios tested, except for 0.12:1 (99% ± 0.5), 0.06:1 (49% ± 0.2), and 0.03:1 (24% ± 1.2). To further investigate the stability of the CNPs-pDNA complex, the polyplexes were incubated in DMEM at different pH values. The results demonstrated that pDNA was released from CNPs when incubated at pH values of 5 and 4, as evidenced by the migration of the pDNA through the agarose gel (Figure 3B).

### 2.3. DNase I Protection Assay of CNPs-pDNA Polyplexes

**Figure 4:**
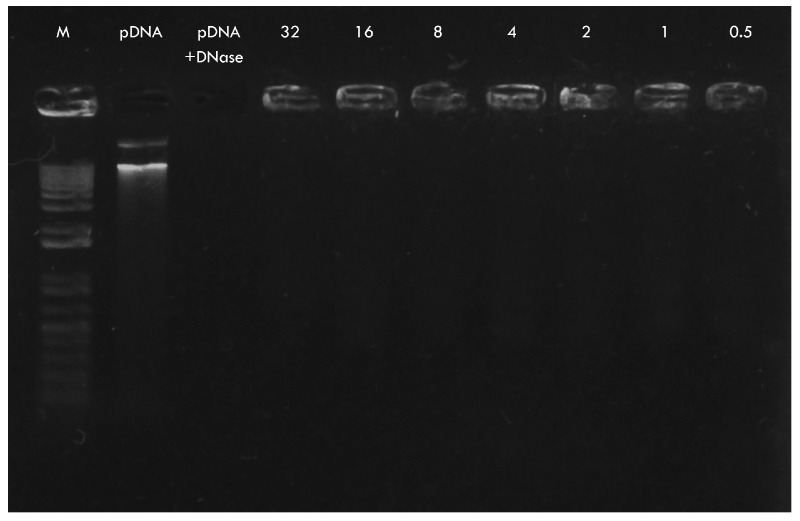
Protection of plasmid DNA by CNPs against DNase I degradation. Agarose gel electrophoresis showing the results of the DNase I protection assay on CNPs-pDNA polyplexes prepared at different C/P weight ratios (32:1 to 0.5:1). M: 1 kb DNA ladder (Invitrogen, 10787018); pDNA: plasmid DNA.

To evaluate the protective capability of CNPs against enzymatic degradation, a DNase I protection assay was performed on CNPs-pDNA polyplexes formed at different C/P weight ratios. In the DNase digestion control (naked pDNA), complete degradation was observed, as no signal was detected on the agarose gel. In contrast, polyplexes formed with CNPs successfully protected the pDNA at all of the tested ratios, including as low as 0.5:1. This was evidenced by the presence of DNA retained in the wells, indicating that the DNA remained intact and bound to the nanoparticles (Figure 4). These results confirm that CNPs provide effective protection against nuclease activity, even at low C/P ratios, which is critical for maintaining plasmid stability during gene delivery.

### 2.4. Cell Viability Assay by MTT

**Figure 5:**
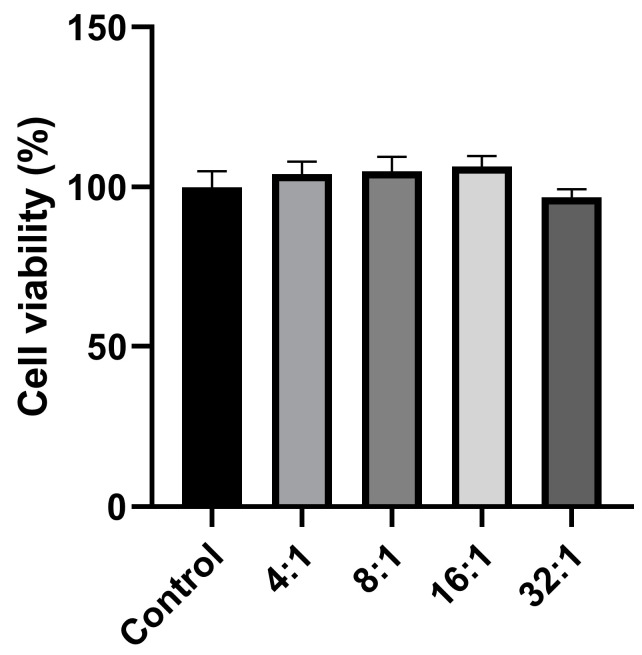
MTT assay for cell viability of HEK-293T cells exposed to CNPs-pDNA polyplexes. Cell viability was measured 24 h after exposure to different C/P weight ratios (4:1, 8:1, 16:1, and 32:1). No significant differences in cell viability were observed between the treated groups and the untreated control, indicating that the CNPs-pDNA polyplexes did not affect cell viability at the tested concentrations. Values are shown as means ± SEM. *p* > 0.05.

The biocompatibility of CNPs-pDNA polyplexes was evaluated by the MTT assay in HEK-293T cells after 24 h of exposure to different C/P weight ratios (4:1, 8:1, 16:1, and 32:1). As shown in Figure 5, all of the tested ratios exhibited cell viabilities comparable to the untreated control, indicating that the CNPs-pDNA polyplexes did not affect cell viability at the tested concentrations. Statistical analysis revealed no significant differences among the groups (*p* > 0.05). These findings support the biosafety and compatibility of unmodified chitosan nanoparticles for gene delivery applications.

### 2.5. Characterization of CNPs-pDNA Polyplexes

**Figure 6:**
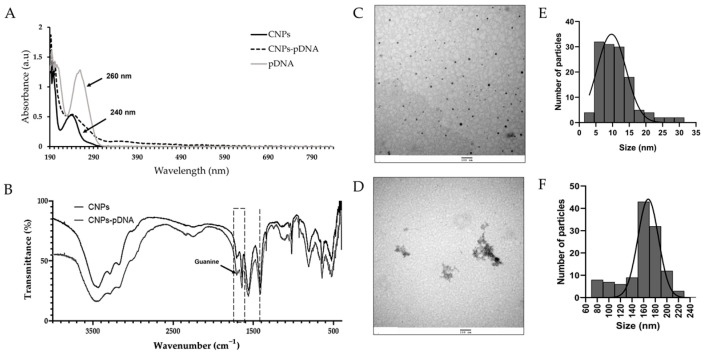
Characterization of CNPs and CNPs-pDNA polyplexes. (**A**) UV-Vis absorption spectra, showing characteristic peaks at 240 nm for CNPs and at 260 nm for DNA. (**B**) FT-IR spectra of CNPs (black) and CNPs-pDNA (gray), with the dashed line representing cytosine (1425 cm^−1^), and the dashed box highlighting the spectra of adenine (1615 cm^−1^), thymine (1661 cm^−1^), and guanine (1684 cm^−1^). (**C**) Representative TEM images of CNPs and (**D**) CNPs-pDNA polyplexes. (**E**) Particle size distribution plot of CNPs based on TEM measurements (n: 130). (**F**) Particle size distribution plot of CNPs-pDNA polyplexes based on TEM measurements (n: 120). Scale bars represent 100 nm.

The physicochemical properties, size, morphology, PDI (polydispersity index), and charge of the CNPs-pDNA polyplexes were characterized. The UV–Visible spectrum of the polyplexes revealed two main absorption peaks: one at 240 nm, corresponding to the CNPs, and another peak at 260 nm, corresponding to pDNA. The spectrum of the polyplexes showed a combined absorption peak in the range of 240–280 nm, indicating the successful formation of CNPs-pDNA polyplexes (Figure 6A). FT-IR analysis of the synthesized CNPs and CNPs-pDNA polyplexes identified several characteristic functional groups (Figure 6B). The broad band around 3500–3350 cm^−1^ corresponded to the stretching vibrations of the –OH and NH_2_ groups in CNPs, while the C–H bending was observed at 2900 cm^−1^. The absorption peaks at 1700–1550 cm^−1^ were attributed to primary and secondary amides, and the peak at 1390 cm^−1^ corresponded to C-N stretching. The signal at 950 cm^−1^ was associated with C–O stretching, and a small signal from guanine in pDNA was observed at 1710 cm^−1^. Transmission electron microscopy (TEM) analysis revealed that the CNPs were spherical with a mean particle size of 11.1 ± 5.2 nm (Figure 6C), while the pDNA polyplexes exhibited a non-homogeneous shape with an increased size of 159.0 ± 33.1 nm (Figure 6D). The size distribution plots derived from the TEM measurements showed a narrow distribution for CNPs, with most particles ranging between 5 and 20 nm (Figure 6E). In contrast, CNPs-pDNA polyplexes displayed a broader size distribution, reflecting the presence of aggregates and variability in particle morphology, which is consistent with the complexation between chitosan and pDNA (Figure 6F).

Dynamic light scattering (DLS) analysis revealed that the hydrodynamic diameter of CNPs was 397.6 ± 51.6 nm, while that of CNPs-pDNA polyplexes increased to 422.1 ± 12.4 nm, suggesting the successful complexation of pDNA with CNPs. Both formulations showed a relatively high PDI of 0.6 ± 0.1, indicating a broad size distribution. The surface charge (zeta potential) of CNPs was +20.4 ± 0.6 mV and slightly decreased to 19.7 ± 0.3 mV after pDNA binding (Table 1).

### 2.6. Transfection of HEK-293T Cells

**Figure 7:**
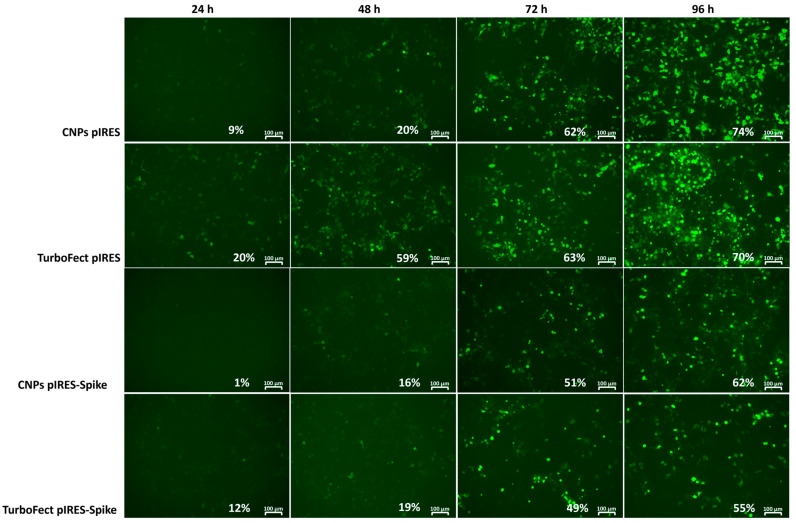
Time course of transfection efficiency in HEK-293T cells using CNPs or TurboFect. Fluorescence microscopy images were captured at 24, 48, 72, and 96 h post-transfection using either the control pIRES2-eGFP or the recombinant pIRES2-eGFP-Spike plasmid, delivered with CNPs or control transfection reagent TurboFect. Transfection efficiency is shown as the percentage of GFP-positive cells. Scale bars = 100 μm.

The in vitro transfection efficiency of CNPs-pDNA polyplexes (C/P weight ratio 8:1) was evaluated in HEK-293T cells using the unmodified pIRES2-eGFP plasmid as a control and the recombinant pIRES2-eGFP-Spike plasmid. TurboFect was employed as a positive control. Transfection was monitored at 24, 48, 72, and 96 h post-transfection via fluorescence microscopy (Figure 7).

TurboFect induced early fluorescence, detectable as early as 24 h post-transfection, with transfection efficiencies of 20% (pIRES2-eGFP) and 12% (pIRES2-eGFP-Spike). In contrast, fluorescence from CNPs was not detected until 48 h post-transfection, with transfection efficiencies of 20% and 16%, respectively. However, by 72 h, both TurboFect and CNPs showed comparable efficiencies (63% vs. 62% for pIRES2-eGFP; 49% vs. 51% for pIRES2-eGFP-Spike). By 96 h, CNPs slightly outperformed TurboFect, with 74% vs. 70% for pIRES2-eGFP and 62% vs. 55% for pIRES2-eGFP-Spike. Notably, fluorescence intensity was higher for the unmodified pIRES2-eGFP plasmid compared to the pIRES2-eGFP-Spike.

### 2.7. Spike mRNA Expression in HEK-293T Cells by RT-qPCR

**Figure 8:**
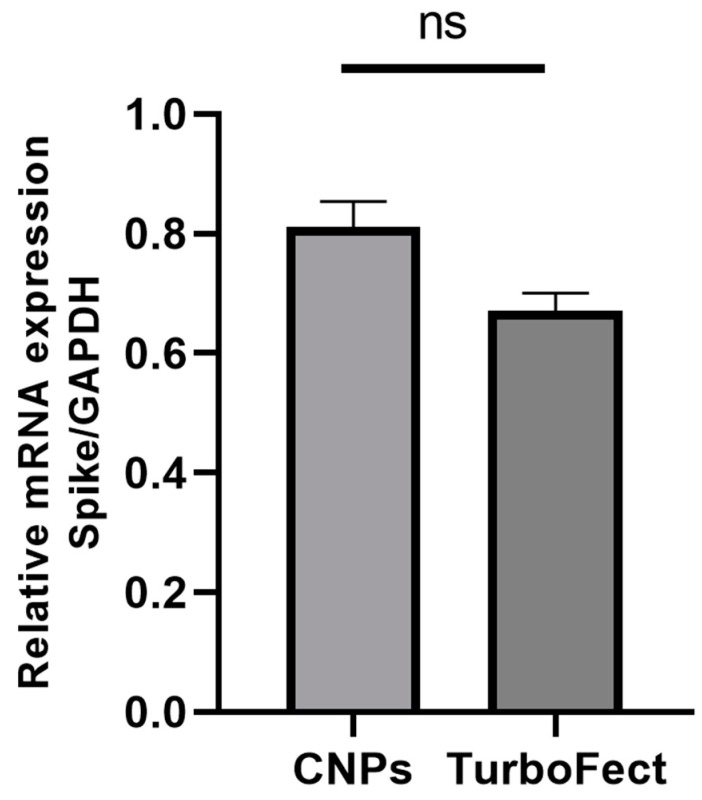
Spike mRNA expression in HEK-293T cells 72 h post-transfection. Relative mRNA levels of the Spike gene were quantified 72 h post-transfection with the pIRES2-eGFP-Spike plasmid using either CNPs or the control reagent TurboFect. Gene expression was normalized to GAPDH and presented as mean ± SEM. Statistical analysis indicated no significant difference between groups (ns, *p* > 0.005). Three biological replicates were analyzed.

The expression of Spike mRNA was evaluated in HEK-293T cells 72 h after transfection with the constructed pIRES2-eGFP-Spike plasmid, delivered either by CNPs or TurboFect. The relative gene expression was analyzed using GAPDH as the housekeeping gene. Although cells transfected with CNPs showed slightly higher Spike mRNA expression compared to those transfected with TurboFect, the difference was not statistically significant (*p* = 0.2) (Figure 8).

### 2.8. Verification of Spike Protein Expression in HEK-293T Cells by Western Blot

**Figure 9:**
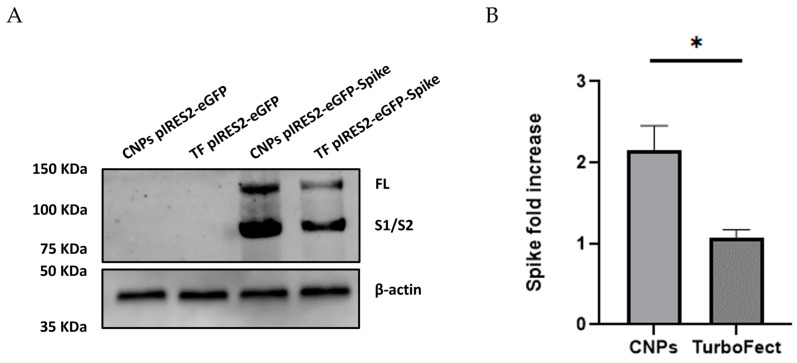
Spike protein expression in transfected HEK-293T cells. (**A**) Western blot analysis of protein extracts from HEK-293T cells collected 96 h post-transfection with either recombinant pIRES2-eGFP-Spike (using CNPs or TurboFect) or control plasmid pIRES2-eGFP. Bands corresponding to full-length Spike protein (~140 kDa) and the S1/S2 subunits (~80 kDa) were detected only in cells transfected with pIRES2-eGFP-Spike. β-actin (~42 kDa) was used as a loading control. CNPs: Chitosan nanoparticles; TF: TurboFect; FL: full-length Spike protein; S1/S2: subunits 1 and 2 from Spike protein. (**B**) Densitometric quantification of Spike expression normalized to β-actin. Data are presented as mean ± SEM. * *p* < 0.05 indicates statistically significant difference.

To confirm the expression of the Spike protein, Western blot analysis was performed on protein lysates from HEK-293T cells 96 h post-transfection with the recombinant pIRES2-eGFP-Spike plasmid delivered via CNPs or TurboFect. The unmodified pIRES2-eGFP plasmid was used as a negative control. Only cells transfected with pIRES2-eGFP-Spike displayed bands corresponding to the full-length Spike protein (FL, ~140 kDa) and its S1/S2 subunits (~80 kDa) (Figure 9A).

While both delivery systems resulted in detectable Spike expression, the signal intensity was notably higher in cells transfected with CNPs. Densitometric analysis, normalized to β-actin (42 kDa), revealed that the Spike protein expression in the CNP group was 2.21-fold higher compared to the TurboFect group (1.01-fold), with this difference being statistically significant (*p* = 0.006) (Figure 9B).

## 3. Discussion

The global health crisis caused by the SARS-CoV-2 pandemic has underscored the urgent need for innovative vaccine technologies. Among the various platforms explored, DNA vaccines have emerged as a promising option due to their ease of production, cost-effectiveness, and stability compared to other platforms, such as RNA-based vaccines [30,31]. However, the immunogenicity of naked DNA vaccines is often limited by their low transfection efficiency, necessitating the use of effective delivery systems [32,33].

Although chitosan nanoparticles have been widely recognized as a promising gene carrier [34], several challenges hinder their effectiveness, such as low transfection efficiency compared to viral vectors, or inefficient endosomal escape. Recent advances in ligand-conjugated chitosan (e.g., folate, RGD peptides) or hybrid systems (e.g., chitosan-PEG, chitosan–lipid hybrids) have improved targeting and cellular uptake [15,26,35].

While chemical and physical modifications of chitosan—such as conjugation with PEG, lipids, or targeting ligands—have been widely explored to improve solubility, enhance the encapsulation of hydrophobic molecules, and stabilize nanoparticle formulations, these strategies also introduce challenges related to reproducibility, scalability, and regulatory approval. These modifications were originally pursued to address the intrinsic limitations of native chitosan, including its low solubility at neutral pH, variable colloidal stability, and reduced efficiency in delivering certain types of genetic material.

For example, functionalized systems have demonstrated improved mucosal targeting (e.g., gold-nanostar chitosan for SARS-CoV-2 DNA vaccines) [36], enhanced antiviral drug delivery [37], and increased permeability in oral delivery using piperine-loaded chitosan-coated lipid nanoparticles [38]. Additionally, tunable systems like chitosan–TPP nanoparticles [39] and hybrid nanomaterials [34,40] have expanded the scope of delivery for nucleic acids and other therapeutics.

However, these approaches often compromise the inherent advantages of chitosan itself, such as its immunomodulatory capacity, biodegradability, and ease of production, while also increasing formulation complexity and production costs. Recent studies have revisited unmodified chitosan systems, demonstrating that they can effectively serve as immune adjuvants [41], and even agents for wound healing and metabolic regulation [40], without the need for complex functionalization. Chitosan’s natural ability to enhance antigen uptake and activate antigen-presenting cells has also been recognized in recent reviews exploring its role as a vaccine adjuvant [42].

These findings collectively highlight a growing recognition of the benefits of simpler, unmodified chitosan systems, especially in the context of DNA vaccine delivery, where cost, stability, and immune activation are key considerations.

In this study, we evaluated the potential of chitosan nanoparticles (CNPs) as a delivery vehicle for plasmid DNA (pDNA), using unmodified chitosan to offer a simpler and more cost-effective alternative while achieving adequate transfection efficiency. We constructed a recombinant plasmid encoding the Spike gene from the BA.1 Omicron subvariant and formed polyplexes with CNPs to transfect HEK-293T cells, subsequently measuring mRNA and protein expression.

The recombinant plasmid encoding the Spike gene from the BA.1 Omicron subvariant was successfully constructed using overlapping RT-PCR fragments and cloned into the pIRES2-eGFP vector (Figure 1). Validation by restriction enzyme digestion and sequencing confirmed the correct insertion and orientation of the Spike gene (Figure 2), ensuring the plasmid’s suitability for downstream transfection experiments.

CNPs were synthesized via ionic gelation, a straightforward and efficient method that leverages the electrostatic interactions between the positively charged chitosan and the negatively charged phosphate groups of pDNA [43].

Gel retardation and release assays confirmed the effective binding of pDNA to CNPs and demonstrated pH-dependent DNA release at endosomal pH levels (Figure 3), supporting their potential for intracellular delivery and endosomal escape. Furthermore, the DNase I protection assay (Figure 4) demonstrated that CNPs effectively protected plasmid DNA from enzymatic degradation across all of the tested C/P ratios (from 0.5:1 to 32:1). In contrast, naked pDNA was completely degraded under the same conditions, as evidenced by the absence of detectable bands. These findings confirm that the electrostatic interactions between chitosan and DNA form stable polyplexes that can resist nuclease attack, a critical feature for successful in vivo delivery.

In addition to their protective capacity, the safety profile of the CNPs-pDNA polyplexes was evaluated through an MTT assay in HEK-293T cells (Figure 5). All of the tested C/P ratios (4:1 to 32:1) exhibited cell viabilities comparable to the untreated control group. These results demonstrate that the CNPs-pDNA polyplexes are safe under the tested conditions, reaffirming the biocompatibility of unmodified chitosan nanoparticles. Our findings are consistent with previous studies demonstrating the safety of chitosan-based nanoparticles for biomedical applications. Trejo-Santillán et al. [44] evaluated the biosafety of conjugated chitosan nanoparticles (chitosan–Protoporphyrin IX–Vitamin B9) and established a safe concentration of 0.25 mg/mL for in vitro applications. Notably, the highest C/P ratio tested in our study (32:1) corresponds to a chitosan concentration of approximately 0.24 mg/mL, remaining within the safety margin reported by Trejo-Santillán et al. This favorable safety profile is essential for gene delivery applications and supports the use of CNPs as a viable alternative to more complex or chemically modified delivery systems.

The UV–Visible spectrum of CNPs and CNPs-pDNA polyplexes revealed an absorption peak at 240 nm, consistent with the presence of the CO group [45]. These findings align with previous reports, confirming the successful formation of CNPs and their polyplexes [46].

Fourier-transform infrared spectroscopy (FT-IR) analysis further supported the formation of CNPs-pDNA polyplexes. Chitosan exhibits characteristic peaks at 1657 cm^−1^ (NH2 vibration), 1564 cm^−1^ (carbonyl-stretching vibration), and 1070–1029 cm^−1^ (C-O stretching vibrations of the pyranose ring) [47], while DNA displays absorbance bands at 1425 cm^−1^ (cytosine), 1615 cm^−1^ (adenine), 1661 cm^−1^ (thymine), and 1684 cm^−1^ (guanine) [48]. The overlap of these peaks in the CNPs-pDNA polyplexes confirmed the successful formation of the complexes, as previously reported [46,49].

Transmission electron microscopy (TEM) revealed that CNPs exhibited spherical morphology, while CNPs-pDNA polyplexes displayed a more heterogeneous structure. Interestingly, studies have shown that globular or aggregated structures, as observed in our polyplexes, can enhance gene delivery efficiency [50]. The dynamic light scattering (DLS) measurements indicated larger hydrodynamic diameters compared to TEM, as DLS accounts for the hydration sphere surrounding the nanoparticles [51,52]. Specifically, the TEM results show the size of the dry particles, while DLS captures the overall diameter, which includes water and other components that may be associated with the particles in solution. The narrower size distribution observed by TEM may reflect the collapse of this hydrated layer under vacuum conditions, emphasizing the differences between dry-state and solution-state measurements. This discrepancy is consistent with previous findings and highlights the importance of using complementary techniques for comprehensive nanoparticle characterization.

The cationic nature of chitosan facilitates cell internalization by interacting with the negatively charged DNA [52]. The zeta potential measurements confirmed the positive surface charge of CNPs (20.4 ± 0.6 mV) and CNPs-pDNA polyplexes (19.7 ± 0.3 mV), which is crucial for preventing aggregation, promoting endosomal escape, and ensuring stability in the extracellular environment [53,54]. However, excessively high positive charges can hinder gene transfection due to strong interactions between CNPs and pDNA, while neutral or negative charges reduce stability [53].

The UV–Vis spectra, FT-IR analysis, and TEM confirmed successful nanoparticle formation and plasmid complexation (Figure 6), while the DLS and zeta potential measurements provided complementary data on size distribution and surface charge (Table 1).

In this study, CNPs-pDNA polyplexes successfully delivered the recombinant Spike gene from SARS-CoV-2. To evaluate their efficiency, we compared CNPs to the commercial transfection reagent TurboFect, a cationic polymer-based system that, like chitosan nanoparticles, facilitates DNA condensation and cellular uptake via electrostatic interactions, making it a relevant comparator [53]. Since the effectiveness of gene therapy can be measured in terms of transfection efficiency [53], GFP and Spike expression in HEK-293T cells confirmed the functionality of the constructed plasmid. Transfection efficiency was comparable between CNPs and TurboFect at 96 h post-transfection, with CNPs showing a slight advantage (62–74% compared to 55–70%) (Figure 7). The delayed GFP expression observed with CNPs may be attributed to the large size of the Spike gene and the sequential translation of GFP, as well as the strong interactions between high-molecular-weight chitosan and DNA, which can slow down gene release since it has enhanced stability and better protection in the endosomal/lysosomal compartments [43]. Additionally, the pH of the transfection media plays a critical role; it has been shown that pH < 6.8 causes strong electrostatic interactions between the negatively charged DNA and the positively charged chitosan, which slows down DNA release [55]. However, Nimesh et al. [56] reported higher transfection efficiency in HEK-293T cells at pH 6.5, suggesting that pH optimization is context-dependent.

Transfection efficiency is influenced by factors such as chitosan molecular weight, degree of deacetylation, pH, and N/P ratio [55]. Since the chitosan used in the present study does not have a defined molecular weight, we propose that the interplay of the aforementioned factors, such as charge and solubility, may collectively contribute to the observed transfection efficiency. Given that the molecular weight of the chitosan was not determined, the N/P ratio could not be calculated. As an alternative, the chitosan–DNA (C/P) ratio was estimated based on weight and subsequently analyzed by agarose electrophoresis. Our results suggest that a C/P ratio of 8:1 is optimal for high transfection efficiency, consistent with previous studies [46,57]. Cao et al. suggest that an N/P rate <25 is more effective for transfection [43]. While many studies report transfection efficiencies ≤40% with pDNA [56,58,59,60,61], our findings align with those of Çelik et al. [62], who achieved 67% efficiency using phosphoryl amine-modified chitosan, and Gao et al. [63], who reached 98% efficiency with quaternized chitosan functionalized with poly (β-amino ester). However, these methods are time-consuming and costly compared to our approach.

The functionality of the constructed plasmid was confirmed by both Spike mRNA and protein expression using RT-qPCR and Western blot analysis. While no significant difference in mRNA expression was observed between CNPs and TurboFect (*p* = 0.2) (Figure 8), CNPs yielded significantly higher protein expression (*p* = 0.006) at 96 h post-transfection (Figure 9). Although chitosan nanoparticles (CNPs) and TurboFect exhibited comparable transfection efficiencies in terms of GFP-positive cells, a notably higher expression of the Spike protein was observed in cells transfected with CNPs. This suggests that CNPs may improve the intracellular retention and stability of pDNA, resulting in more sustained protein expression. In contrast, TurboFect could form fewer stable complexes and tend to release pDNA more rapidly after cellular uptake, which might lead to a shorter window of protein expression. The delayed expression observed with CNPs could be advantageous for vaccine applications, as it may prolong immune stimulation and reduce the need for booster doses.

The COVID-19 pandemic has highlighted the need for innovative vaccine technologies. Chitosan, a biocompatible polysaccharide, has shown promise as a pDNA delivery vehicle [34,43,53,64] due to its immune-stimulating properties, such as enhancing macrophage activation, cytokine production, and cytotoxic T lymphocyte (CTL) responses [65,66]. Nanomaterials like CNPs offer several advantages over naked DNA, including improved delivery to lymphoid tissues, enhanced transfection efficiency, and the facilitation of dendritic cell maturation and antigen presentation [67,68]. These properties make CNPs a promising platform for DNA vaccine delivery. However, to better understand the translational potential of the CNP-based gene delivery platform proposed in this study, further research is needed to assess its in vivo biodistribution, long-term expression dynamics, and ability to elicit protective immune responses. Future studies should include animal models to evaluate tissue-specific accumulation, the persistence of gene expression, and immunological outcomes such as antibody production and T cell activation. Such investigations would provide essential data to validate CNPs as a clinically relevant DNA vaccine delivery system.

## 4. Materials and Methods

### 4.1. Construction of DNA Plasmid pIRES2-eGFP-Spike

#### 4.1.1. Synthesis of Spike cDNA by RT-PCR from Positive Samples

The recombinant plasmid pIRES2-eGFP-Spike encodes an unmodified SARS-CoV-2 Spike protein derived from the Omicron variant. It was constructed using the mammalian expression vector pIRES2-eGFP (Takara Bio USA, San Jose, CA, USA) as the backbone. The sequence encoding the Spike protein was obtained by RT-PCR from positive RNA samples of subvariant BA.1, provided by the Biobank of the Instituto Mexicano del Seguro Social (IMSS). The RNA concentration was measured at A260/A280 nm using a Nanodrop 2000 spectrophotometer (Thermo Scientific, Waltham, MA, USA). Complementary DNA (cDNA) synthesis was performed using the RevertAid cDNA First Strand Synthesis Kit (Thermo Fisher Scientific, Waltham, MA, USA; cat. #K1622, originally developed by Fermentas), with specific primers (Reverse F1, 5′ *ACACCCTGATAAAGAACAGC* 3′ and Reverse F2, 5′ *TTGATTTCACCTTGCTTCAAAGTTAC* 3′). Briefly, 400 ng of RNA pretreated with DNase I (1 U) was used for cDNA synthesis under the following conditions: 42 °C for 60 min, followed by 70 °C for 5 min. The resulting cDNA was diluted in DNAse/RNAse free water and stored at −20 °C until use.

#### 4.1.2. Amplification of the Spike Sequence

The complete Spike sequence was obtained in two steps. First, two pairs of primers were used to amplify the gene in two separate fragments (F1 and F2) using PCR with Q5 high-fidelity DNA polymerase (New England Biolabs, Ipswich, MA, USA; catalog #M0491S) and the previously synthesized cDNA as a template. For the F1 fragment, the primer set used was 5′ *AGGGGTACTGCTGTTATGTC* 3′ as primer forward, and 5′ *ACACCCTGATAAAGAACAGC* 3′ for primer reverse. For the F2 fragment, the primer set was 5′ *TCCAACAATTTGGCAGAGAC* 3′ as primer forward, and 5′ *TTGATTTCACCTTGCTTCAAAGTTAC* 3′ for primer reverse. To introduce restriction enzyme recognition sites for NheI and SacI by PCR-directed mutagenesis, the primers for F1 and F2 were modified. For F1, the forward primer was replaced with 5′ *TGTTCTT**GCTAGC**AACTAAACGAAC* 3′ (NheI site underlined), and for F2, the reverse primer was changed to 5′ *ATCCAT**GAGCTC**GTTTATGTGTAATG* 3′ (SacI site underlined). The modified F1 and F2 fragments were then joined using Overlap and Extension Touchdown PCR (POE-T) with Platinum SuperFi II DNA Polymerase (Thermo Fisher Scientific, Waltham, MA, USA; catalog #12361010). The accuracy of the overlap between F1 and F2 fragments was confirmed by sequencing the POE-T product using the primer from Table 2. The product was sent to the *Instituto de Fisiología* at UNAM for Sanger sequencing.

#### 4.1.3. Cloning of the Spike Sequence into pIRES2-eGFP

The resulting fragment obtained from the POE-T process was digested with the restriction enzymes NheI (Thermo Fisher Scientific, Waltham, MA, USA; cat. #FD0973) and SacI (Thermo Fisher Scientific, Waltham, MA, USA; cat. #FD1133) and then cloned into the previously digested pIRES2-eGFP vector. The digestion products were purified with the PureLink™ Quick Gel Extraction and PCR Purification Combo Kit (Thermo Fisher Scientific, Waltham, MA, USA; cat. #K220001) and ligated with T4 DNA Ligase (Thermo Fisher Scientific, Waltham, MA, USA; cat. #EL0011). The resulting plasmid, named pIRES2-eGFP-Spike, was used to transform competent *E. coli* DH5 cells, which were then grown on LB Broth agar (Sigma-Aldrich, St. Louis, MO, USA; cat. #L3022, L3022) plates containing 100 mg/mL kanamycin (Sigma-Aldrich, St. Louis, MO, USA; cat. #K1876). Transformants were screened by restriction analysis combined with PCR and confirmed by sequencing. The recombinant plasmid was produced in *E. coli* DH5α and purified using the EndoFree Plasmid Mega Kit (QIAGEN, Hilden, Germany; cat. #12381).

### 4.2. Synthesis of Chitosan Nanoparticles and Preparation of pDNA Polyplexes

Chitosan nanoparticles (CNPs) were synthesized using ≥75% deacetylated chitosan, with no specific molecular weight (Sigma-Aldrich, St. Louis, MO, USA; cat. #C3646-25G), following the method described by Mendoza-Guevara et al. [46] with minor modifications. Briefly, to form CNPs-pDNA polyplexes through electrostatic interactions, the constructed plasmid DNA (pIRES2-eGFP-Spike) was added immediately after the addition of 3 µL of sodium phosphate 1 M (J.T. Baker, Ciudad de México, Mexico; cat. #3821), and 30 µL of chitosan solution (1 mg/mL) prepared with 0.5% acetic acid. To enhance positive charges, 1 µL of a chitosan solution (1 mg/mL) prepared with 40% acetic acid and ethylenediamine (Sigma-Aldrich, St. Louis, MO, USA; cat. #E26266) adjusted to pH 5 was added and then brought to a final volume of 50 µL for use in each well. The mixture was vortexed and incubated at room temperature for 15 min to facilitate polyplex formation. Both CNPs and CNPs-pDNA polyplexes were prepared fresh before use.

### 4.3. Retardation and Release Assay of pDNA Polyplexes

The binding ability of CNPs and pDNA was evaluated using agarose gel electrophoresis at various Chi/pDNA (C/P) weight (μg/μg) ratios (0.03:1–32:1). The polyplexes were mixed with 10x BlueJuice (Thermo Fisher Scientific, Waltham, MA, USA; Invitrogen™, cat. #10816015) and SYBR^TM^ Gold (Thermo Fisher Scientific, Waltham, MA, USA; Invitrogen™, cat. #S11494) and loaded onto a 1% agarose gel with TBE buffer. Electrophoresis was conducted at 100 mV for 60 min, and the gel was visualized using an ImageQuant™ LAS 4000 biomolecular imager (GE Healthcare, Chicago, IL, USA).

The reversibility of the CNPs-pDNA association was assessed by incubating the polyplexes in DMEM adjusted to different pH values (4–10) for 10 min. The release of pDNA was then analyzed by agarose gel electrophoresis as described above.

### 4.4. DNase I Protection Assay of CNPs-pDNA Polyplexes

To evaluate the protective capability of CNPs against enzymatic degradation, a DNase I protection assay was performed on CNPs-pDNA polyplexes formed at different C/P weight ratios (0.5:1–32:1). For each ratio, 4 µg of pDNA was complexed with the corresponding amount of CNPs. Aliquots of the polyplexes (8 µL, containing approximately 640 ng of pDNA) were then treated with 1 U (1 µL) of DNase I (Thermo Fisher Scientific, Waltham, MA, USA; cat. #EN0521) in 1 µL of DNase reaction buffer (10 mM Tris-HCl, 2.5 mM MgCl_2_, 0.5 mM CaCl_2_, pH 7.6) and incubated at 37 °C for 30 min. The enzymatic reaction was terminated by adding 1 µL of 0.5 M EDTA and incubating at 65 °C for 10 min to inactivate the enzyme. The samples were analyzed by electrophoresis on a 1% agarose gel stained with SYBR™ Gold. Naked pDNA treated with DNase I was included as a control for complete degradation, while untreated pDNA served as a negative control.

### 4.5. Cell Viability Assay by MTT

Immortalized human embryonic kidney (HEK-293T) cells from ATCC (CRL-3216) were generously provided by Dra. Sonia Mayra Pérez-Tapia. HEK-293T cells were cultured in DMEM (Gibco, 12800017) supplemented with 10% fetal bovine serum (Gibco, 26140079) and 1% antibiotics (100 U/mL penicillin and 100 μg/mL streptomycin), reaching 80% confluence. Cell cultures were maintained at 37 °C in a humidified incubator with 5% CO_2_.

HEK-293T cells were seeded in 96-well plates at a density of 5 × 10^3^ cells per well and incubated for 24 h at 37 °C with 5% CO_2_. After incubation, the culture medium was replaced with fresh DMEM containing CNPs-pDNA polyplexes formed at different C/P weight ratios (4:1 to 32:1). Cells treated with DMEM alone served as the negative control.

After 24 h of exposure, cell viability was assessed using the MTT assay. Cells were incubated with DMEM containing 100 μg/mL MTT (Sigma-Aldrich, St. Louis, MO, USA) for 4 h at 37 °C. Subsequently, the medium was removed, and the formazan crystals that formed were dissolved in acidic isopropyl alcohol (pH 4). Absorbance was measured at 595 nm using a microplate reader ELISA ELx808 (Biotek Instruments, Winooski, VT, USA). Cell viability was expressed as a percentage relative to the untreated control group. Each treatment was performed in triplicate, and the results were expressed as the mean ± standard deviation.

(1) $$\%\;Viability=\frac{Mean\;absorbance\;of\;ALA\;treated\;cells}{Mean\;absorbance\;of\;control\;cells}\times 100$$

### 4.6. Characterization of CNPs-pDNA Polyplexes

#### 4.6.1. Spectroscopic Measurement by UV-Vis

The optical absorption spectrum of the CNPs-pDNA polyplexes was obtained using ultraviolet–visible (UV-Vis) spectroscopy. For this analysis, 2 μL of the polyplexes was analyzed with a NanoDrop 2000 spectrophotometer (Thermo Fisher Scientific, Wilmington, DE, USA). The scan was performed over a wavelength range from 200 to 800 nm.

#### 4.6.2. Chemical Composition by Fourier-Transform Infrared Spectroscopy (FT-IR)

Approximately 1 mL of the CNPs-pDNA polyplexes sample was lyophilized using a Labconco lyophilizer (FreeZone™ Freeze-Dry Systems, Kansas City, MO, USA) for 8 h. The lyophilized sample was then processed using the potassium bromide (KBr) tablet method. The resulting tablets were analyzed with an FT-IR Spectrometer (Thermo Fisher Scientific, Nicolet 6700, Waltham, MA, USA).

#### 4.6.3. Size and Morphology by Transmission Electron Microscopy (TEM)

The morphology and size of the nanoparticles were examined using a JEM-1010 transmission electron microscope (JEOL Ltd., Tokyo, Japan) operating at 60 kV. A 200-mesh copper grid was immersed in 50 μL of the sample for 15 min. Negative staining was performed by immersing the grid in 4% alcoholic uranyl acetate for 15 min. The grid was then washed with Milli-Q water to remove excess reagent and allowed to dry before imaging.

#### 4.6.4. Size, PDI, and Zeta Potential by Dynamic Light Scattering (DLS)

The hydrodynamic diameter, polydispersity index (PDI), and zeta potential of the CNPs-pDNA polyplexes were measured using DLS with a Zetasizer Nano-S90 (Malvern Panalytical, Malvern, UK). Briefly, 1 mL of the polyplex sample was placed in a cuvette, and triplicate measurements were performed to obtain the average values for diameter, PDI, and zeta potential. The results were expressed as mean ± standard deviation (SD).

### 4.7. Transfection of HEK-293T Cells

HEK-293T cells were seeded on a 24-well plate at an initial density of 5 × 10^5^ cells per well and incubated overnight at 37 °C with 5% CO_2_. Transfection was performed after 24 h, once the cells had adhered and reached optimal confluency. For transfection, 4 μg of either the control plasmid pIRES2-EGFP or the constructed plasmid pIRES2-eGFP-Spike was mixed with 32 μg of CNPs (1 μg/μL) or with the transfection reagent TurboFect (Thermo Fisher Scientific, Waltham, MA, USA; cat. #R0533). Prior to transfection, the old cell medium was replaced with fresh supplemented DMEM with 10% FBS (for wells transfected with CNPs-pDNA) or Opti-MEM (Thermo Fisher Scientific, Waltham, MA, USA; Gibco™, cat. #11058021) (for wells transfected with TurboFect polyplexes). Following the addition of the transfection mixtures, the plate was incubated at 37 °C for 24 h. After this period, the cell medium was then replaced with fresh DMEM supplemented with 10% FBS. Cells were monitored at 24, 48, 72, and 96 h post-transfection for fluorescence using a ZOE Fluorescent Cell Imager (Bio-Rad, Hercules, CA, USA). Transfection efficiency was calculated as the percentage of GFP-positive cells relative to the total number of cells counted using ImageJ version 1.54 g for Windows.

#### 4.7.1. Spike mRNA Expression in HEK-293T Cells by RT-qPCR

Total RNA from HEK-293T cells 72 h post-transfection was extracted using Trizol Reagent (Sigma-Aldrich, St. Louis, MO, USA; cat. #T9424). The RNA was dissolved in nuclease-free water and stored at −80 °C until further analysis. The RNA concentration was measured at A260/A280 nm using a Thermo Scientific NanoDrop 2000 spectrophotometer. For cDNA synthesis, 400 ng of RNA, pretreated with DNase I (1 U), was used with the RevertAid cDNA First Strand Synthesis Kit (Thermo Fisher Scientific, Waltham, MA, USA; cat. #K1622), incorporating oligo(dT)18 primers. The resulting cDNA was diluted in DNase/RNAse-free water and stored at −20 °C. Specific primers for Spike were designed using SnapGene software (Dotmatics, version 7.0.2), and GAPDH primers were reported by Demidenko et al. [69] (Table 2). Quantitative PCR (qPCR) was performed with 20 ng of cDNA and 1 μL of SYBR Green II 25x (SEEL Sunshine Laboratories, Beijing) using Platinum Taq Polymerase (Thermo Fisher Scientific, Waltham, MA, USA; Invitrogen™, cat. #10966018) on a StepOne real-time PCR detection system (Applied Biosystems by Thermo Fisher Scientific, Foster City, CA, USA). GAPDH was used as an endogenous control to calculate the relative expression using the 2^−ΔΔCt^ method, comparing CNPs to TurboFect.

#### 4.7.2. Spike mRNA Expression in HEK-293T Cells by Western Blot

HEK-293T cells were seeded in 6-well plates and transiently transfected with 4 μg of either pIRES2-eGFP-Spike or the control plasmid using CNPs or TurboFect transfection reagent. The transfected cells were incubated at 37 °C with 5% CO_2_ for 96 h and then lysed using RIPA protein extraction buffer 5× (BIO WORLD, Dublin, OH, USA; cat. #41820002.1). The protein concentration was determined using the Pierce™ BCA Protein Assay Kit (Thermo Fisher Scientific, Waltham, MA, USA; cat. #23227). Proteins were separated by 10% SDS-PAGE, transferred to a Trans-Blot nitrocellulose membrane (Bio-Rad Laboratories, Hercules, CA, USA; cat. #162-0115), and subjected to Western blot analysis to verify Spike protein expression. The primary antibodies used were rabbit anti-SARS Spike Protein Antibody (Novus Biologicals, Centennial, CO, USA; cat. #NB100-56578) at a 1:500 dilution and anti-beta Actin antibody (Abcam, UK, cat. #ab75186) at a 1:1000 dilution. Detection was performed with a horseradish peroxidase (HRP)-conjugated secondary goat anti-rabbit IgG antibody (Santa Cruz, Dallas, TX, USA; cat. #sc-3837), diluted 1:2000 in tris-buffered saline (TBS). Proteins were visualized using Amersham ECL Prime Western blotting Detection Reagent (Cytiva, Marlborough, MA, USA; formerly GE Healthcare, cat. #28980926) and imaged with an ImageQuant™ LAS 4000 biomolecular imager (GE Healthcare). The protein expression levels were quantified using ImageJ version 1.53e for Windows software.

### 4.8. Statistical Analysis

Experiments were performed in triplicate. The Mann–Whitney U-test was used to analyze the relative mRNA and protein expression levels between the CNPs and TurboFect transfection controls. Statistical analysis was performed using GraphPad Prism 9 (San Diego, CA, USA). A *p*-value of ≤0.05 was considered statistically significant.

## 5. Conclusions

Chitosan nanoparticles (CNPs) have demonstrated significant potential as an effective in vitro delivery system for recombinant plasmid DNA (pDNA), achieving transfection efficiencies comparable to commercial reagents such as TurboFect. Notably, CNPs accomplished this without the need for additional ligands, leveraging the intrinsic properties of chitosan, such as its cationic nature and biocompatibility. This study highlights the CNPs ability to facilitate efficient gene delivery, positioning them as a viable, simple, and cost-effective alternative for enhancing gene-based vaccines and therapeutic applications. By optimizing the physicochemical properties of CNPs and addressing limitations such as transfection efficiency, our work contributes to the expanding body of research on non-viral gene delivery platforms. These findings underscore the potential of CNPs as a versatile platform for the delivery of genetic material, with important implications for advancing gene therapy, vaccine development, and addressing ongoing and future biomedical challenges.

## Figures and Tables

**Table 1:** Physicochemical characterization of CNPs and CNPs-pDNA polyplexes by TEM, DLS, and zeta potential measurements.

Characterization	CNPs	CNPs-pDNA
TEM diameter (nm)	11.1 ± 5.2	159.0 ± 33.1
Hydrodynamic diameter (nm)	397.6 ± 51.6	422.1 ± 12.4
PDI	0.6 ± 0.1	0.6 ± 0.1
Zeta potential (mV)	20.4 ± 0.6	19.7 ± 0.3

Values are expressed as mean ± standard deviation. CNPs: chitosan nanoparticles; pDNA: plasmid DNA; TEM: transmission electron microscopy; PDI: polydispersity index.

**Table 2:** Primers and cycling conditions used for obtaining and validating the Spike sequence by PCR.

Target Sequence (pb)	Primer	Sequence 5′-3′	Cycling Conditions (40 Cycles)
Spike F1 (1974 bp)	Forward F1	AGGGGTACTGCTGTTATGTC	98 °C—15 s 56 °C—15 s 72 °C—120 s
	Reverse F1	ACACCCTGATAAAGAACAGC	
Spike F2 (2208 bp)	Forward F2	TCCAACAATTTGGCAGAGAC	98 °C—15 s 57 °C—15 s 72 °C—120 s
	Reverse F2	TTGATTTCACCTTGCTTCAAAGTTAC	
Spike F1 with NheI site (1850 bp) ^(a)^	Forward S-RE	TGTTCTT**GCTAGC**AACTAAACGAAC	98 °C—15 s 58 °C—20 s 72 °C—120 s
	Reverse F1	ACACCCTGATAAAGAACAGC	
Spike F2 with SacI site (2153 bp) ^(b)^	Forward F2	TCCAACAATTTGGCAGAGAC	98 °C—15 s 58 °C—20 s 72 °C—120 s
	Reverse S-ER	ATCCAT**GAGCTC**GTTTATGTGTAATG	
Sequencing (397 bp)	Forward CMV	GCCCAGTACATGACCTTATGGG	98 °C—15 s 60 °C—15 s 72 °C—20 s
	Reverse CMV	GCCCAGTACATGACCTTATGGG	
qPCR Spike (160 bp)	Forward qPCR-Spike	TAGGGCGTGATCTCCCTCAG	98 °C—15 s 63 °C—17 s 72 °C—15 s
	Reverse qPCR-Spike	TAAGCTGCAGCACCAGCTGT	
qPCR GAPDH (146 bp)	Forward qPCR-GAPDH	AGGTCGGAGTCAACGGATTT	98 °C—15 s 63 °C—17 s 72 °C—15 s
	Reverse qPCR-GAPDH	ATGGGTGGAATCATATTGGAAC	

(a) NheI sequence is shown in underlined text. (b) SacI sequence is shown in underlined text.

## Data Availability

The original contributions presented in the study are included in the article, further inquiries can be directed to the corresponding author.
